# Emergency intraosseous access in a helicopter emergency medical service: a retrospective study

**DOI:** 10.1186/1757-7241-18-52

**Published:** 2010-10-07

**Authors:** Geir A Sunde, Bård E Heradstveit, Bjarne H Vikenes, Jon K Heltne

**Affiliations:** 1 Department of Anaesthesia and Intensive Care, Haukeland University Hospital, Bergen, Norway; 2 Helicopter Emergency Medical Services (HEMS) - Bergen, Norway; 3 Department of Medical Sciences, University of Bergen, Bergen, Norway

## Abstract

**Background:**

Intraosseous access (IO) is a method for providing vascular access in out-of-hospital resuscitation of critically ill and injured patients when traditional intravenous access is difficult or impossible. Different intraosseous techniques have been used by our Helicopter Emergency Medical Services (HEMS) since 2003. Few articles document IO use by HEMS physicians. The aim of this study was to evaluate the use of intraosseous access in pre-hospital emergency situations handled by our HEMS.

**Methods:**

We reviewed all medical records from the period May 2003 to April 2010, and compared three different techniques: Bone Injection Gun (B.I.G^® ^- Waismed), manual bone marrow aspiration needle (Inter V - Medical Device Technologies) and EZ-IO^® ^(Vidacare), used on both adults and paediatric patients.

**Results:**

During this seven-year period, 78 insertion attempts were made on 70 patients. Overall success rates were 50% using the manual needle, 55% using the Bone Injection Gun, and 96% using the EZ-IO^®^. Rates of success on first attempt were significantly higher using the EZ-IO^® ^compared to the manual needle/Bone Injection Gun (p < 0.01/p < 0.001). Fifteen failures were due to insertion-related problems (19.2%), with four technical problems (5.1%) and three extravasations (3.8%) being the most frequent causes. Intraosseous access was primarily used in connection with 53 patients in cardiac arrest (75.7%), including traumatic arrest, drowning and SIDS. Other diagnoses were seven patients with multi-trauma (10.0%), five with seizures/epilepsy (7.1%), three with respiratory failure (4.3%) and two others (2.9%). Nearly one third of all insertions (n = 22) were made in patients younger than two years. No cases of osteomyelitis or other serious complications were documented on the follow-up.

**Conclusions:**

Newer intraosseous techniques may enable faster and more reliable vascular access, and this can lower the threshold for intraosseous access on both adult and paediatric patients in critical situations. We believe that all emergency services that handle critically ill or injured paediatric and adult patients should be familiar with intraosseous techniques.

## Background

Vascular access is important in the resuscitation of critically ill or injured adult and paediatric patients [1,2]. It can be challenging to obtain vascular access, especially in the resuscitation of small children in emergency situations [3-5]. The European Resuscitation Council 2005 guidelines [6] and International Liaison Committee on Resuscitation guidelines [4] recommend intraosseous access during resuscitation if intravenous access proves to be difficult or impossible. Despite these recommendations, intraosseous techniques appear to be rarely used [7]. While numerous reports have been published about the use of different intraosseous devices in emergency patients, they are primarily from paramedic-based ambulance services [2,8]. Few comparisons have been published of different IO techniques used by physicians in emergency departments [7] or in HEMS services manned by physicians/nurses [9,10].

Typical HEMS operating conditions make special demands on medical equipment such as IO devices. Rain, cold, darkness and non-sterile conditions mean that such equipment must be durable and simple to use in all conditions. User friendliness is important for rescuers, both on-scene and in-flight [10].

Intravenous access is traditionally regarded as the optimal route for medication and fluids, and the intraosseous route is often described as the best alternative choice [3,4,11]. Endotracheal, umbilical or intracardial routes are poorer alternatives as regards speed of insertion and reliability in emergency resuscitation. Great saphenous vein cutdown as an emergency surgical approach has also been replaced by the faster IO technique [3,12]. In newborn resuscitation, umbilical venous access is often preferred, with intraosseous as an alternative route [12,13].

Intraosseous technique has been described as a simple and reliable method in both cadaver and clinical studies [9,11,14]. The aim of this study was to evaluate the use of intraosseous access in emergency situations handled by physicians in a pre-hospital HEMS service.

## Methods

Our HEMS helicopter and rapid response vehicle are based at the regional university hospital. The HEMS covers an area of about 15,500 square kilometres of Western Norway, with a population of approximately 500,000. The majority (97%) of missions are 'code red' emergencies [15] and involve medical (65%) and trauma (35%) cases, including incubator transport. During the study period, the HEMS treated 6,116 patients in total, 10.6% of whom were younger than six years.

The HEMS is staffed by six consultants and one registrar. All are experienced anaesthesiologists with extensive knowledge of establishing intravenous access, in both peripheral and central lines, in critically ill patients in emergency situations. As part of their HEMS training programme, intraosseous training was given using manual needles, Bone Injection Guns, and EZ-IO^® ^on both manikins and cadavers. All HEMS physicians have used the technique on patients during resuscitations. The devices were mainly used on-scene before commencing transport, but some insertions were made en route to the hospital (in the helicopter or ambulance) or after arrival at the emergency department. The equipment used in this study has been standard issue for our HEMS service.

Our study population included adult and paediatric emergency patients on whom IO access was performed or attempted by our HEMS unit between May 2003 and April 2010. Data collection was based on a retrospective review of all medical records, and we compared three techniques (B.I.G^®^, Manual needle and EZ-IO^®^) in relation to insertion success rates, insertion-related problems and complications, insertion site, patient age ranges and presenting diagnosis. Age stratification was chosen to differentiate small children, pre-school children, older children and adults (Table 1). Follow up of in-hospital records was done to document complications, needle removal times, antibiotics and outcome.

**Table 1:** IO distribution according to patient age:

Patient age	Number of patients who recieved IO	Total number of patients treated	IO insertion rate %
0-2 years	18	453	3.97%
3-6 years	0	198	0.00%
7-17 years	5	486	1.03%
18-78 years	47	4979	0.94%

IO - Intraosseous.

In the period 2003 to 2006, we used the B.I.G^® ^(Bone Injection Gun - Waismed) for adult patients and a manual bone marrow aspiration needle (Inter-V - Medical Device Technologies) for paediatric patients. Since 2006, we have used the EZ-IO^® ^(Vidacare) for all patients.

This study was not subject to approval by the Regional Committee for Medical Research Ethics but was submitted there for evaluation, and they had no objections to the study or the results being published.

Study data were collected in a separate research database. Rates of success for the different devices were compared using exact Chi-square tests. Contrast between groups for success on first attempt and total success was calculated, and presented with 95% CI.All statistical analyses were performed using SPSS version 17.0 (SPSS Inc., Chicago, IL, USA) and Statistical Analysis System (SAS) version 0.2 software for Windows (SAS Institute, Inc., Cary, North Carolina). Exact confidence intervals were obtained by using the PROF FREQ procedure in SAS. A p-value < 0.05 was considered significant.

## Results

### IO insertion success rates

During the seven-year period, 78 insertion attempts were made on 70 patients. Overall success rates for the different methods were 50% using the manual needle, 55% using the Bone Injection Gun, and 96% using the EZ-IO^®^. Insertion success data for each device are presented in Table 2. Rates of success on first attempt were significantly higher using the EZ-IO^® ^compared to the manual needle/Bone Injection Gun (p < 0.01/p < 0.001). We found no reduction in failure rates over time for each device. Apart from the manual needle (where small numbers are confounding), the B.I.G^® ^showed consistent annual failure rates of 43 to 50% over three years. The EZ-IO^® ^showed failure rates of 5 to 8% in its third and fourth years of service, and zero failure rates in the first, second and fifth years. Insertion failures were equally distributed among the physicians involved.

**Table 2:** Insertion data and success rates with manual needle, B.I.G. and EZ-IO:

IO device	Number of patients who recieved IO	Number of insertions	Success on 1.attempt	Success on 2. attempt	Success on 3. attempt	Failed insertions	First attempt success ** (95%CI)	Overall success *** (95%CI)
Manual needle	5*	6	2	1	0	3	40% (5-85)	50% (12-88)
B.I.G.	18*	22	10	1	1	10	56% (31-79)	55% (32-76)
EZ-IO	49	50	47	1	0	2	96% (86-100)	96% (86-100)

Total number	70*	78	59	3	1	15	84%	81%

Successes on first attempt were compared using exact chi-square test. The contrasts between EZ-IO^® ^and the manual needle/Bone Injection Gun were significant (p < 0.01/p < 0.001). Manual needle vs. B.I.G. was not significant (p = 0.64).

* Two patients had the first attempt with a Manual needle, and the second attempt with a B.I.G.

** First attempt success is calculated using the "Success on 1. attempt" related to "Number of patients who received IO". *** Overall success is calculated using all successful attempts related to "Number of insertions".

IO - Intraosseous.

### Insertion-related problems and complications

Fifteen failures were due to insertion-related problems (19.2%), with four technical problems (5.1%) and three extravasations (3.8%) being the most frequent causes. With the manual needle, we registered one case of needle bending and one case of extravasation. Technical complications such as the bending of needles, malfunction of equipment and misplacement of needles were registered in three cases using the B.I.G^®^. Iatrogenic fracture of the bone at the insertion site with subsequent extravasation happened once with the B.I.G^®^. No technical problems were encountered with the EZ-IO^®^. One accidental dislocation of needle (EZ-IO^®^) was registered in the intensive care unit, and one case of extravasation due to the EZ-IO^® ^being inserted into a traumatic fractured tibia was documented.

### Insertion site

Forty-six of the insertions (59.0%) were made in the proximal tibia. Three were made in the proximal humerus (3.8)%. In the 29 remaining cases, the insertion site was not registered (37.2%).

### Patient age ranges

Patients ranged from one week to 78 years old. Nearly one third of all insertions (n = 22) were made in patients younger than two years. Intraosseous use by age is presented in Table 1.

### Presenting diagnosis

Intraosseous access was primarily used in connection with 53 patients in cardiac arrest (75.7%), including traumatic arrest, drowning and SIDS. Other diagnoses were seven patients with multi-trauma (10.0%), five with seizures/epilepsy (7.1%), three with respiratory failure (4.3%) and two others (2.9%). Intraosseous access was used in 4.8% of all cardiac arrests (n = 1099) and in 1.3% of all non-arrest trauma patients (n = 549). In those younger than two years who received IO, 13 patients (72%) were in cardiac arrest.

### Follow up

Of the 70 patients included, 40 patients (57%) survived to hospital admission and only 12 patients (17%) survived to hospital discharge. Only half of the patients (50%) received antibiotics during the hospital stay as treatment for other medical conditions, despite the recommendation of one prophylactic dose to all IO treated patients. IO needles were removed within two hours of hospital admittance in seven patients. Needle removal times for the remaining patients were not documented. No cases of osteomyelitis or other serious complications were documented during the follow-up of hospital records.

## Discussion

Newer intraosseous techniques such as the EZ-IO^® ^enable faster and more reliable emergency vascular access than the older spring-loaded and manual techniques in our study. In our opinion, this may lower the threshold for using intraosseous access in emergency situations. IO is particular useful in pre-hospital paediatric emergencies where IV access may be impossible.

The small series, especially the low number of manual insertions, and the retrospective design are limitations in our study. Comparison of devices over different time frames may cause bias in interpretation of the results. Nonetheless, as the different techniques were used by the same medical crews, on the same type of patients, and on the same indications - we believe that the differences in time frames do not confound the conclusions. Also, the limited number of physicians involved ensures high reliability in relation to the different techniques used.

All our IO insertions were done by field anaesthesiologists with experience of establishing IV access in emergency patients. Paramedic or nurse-based EMS units often report higher IO insertion rates [2]. Intraosseous technique may be used more frequently for vascular access in less experienced emergency services. The low intervention rate of inserted IO in our study supports this view, and this rate is comparable with results from other physician-staffed HEMS services [9,14,16].

In relation to the Bone Injection Gun, some studies have shown impressive insertion success rates of between 91% and 100% [2,17]. We found consistent low success rates with the B.I.G^®^, with insertion failures equally distributed among the physicians. The rotation of staff and acquired device experience did not seem to influence these results. The overall success rate with the B.I.G^® ^in our material was only 55%, and we believe that this is not good enough when better alternatives are available. Other reports support our finding that physicians achieve lower success rates using this technique [14,18].

Several studies have shown high insertion success rates using the EZ-IO^® ^[8,19], as well as fast and easy insertions [20]. This indicates user friendliness and confirms our results [11]. The development of new powered techniques may increase the rate of successful intraosseous access.

We believe paediatric resuscitation may benefit most from IO use [12]. Intraosseous access compares favourably with umbilical venous catheterisation in newborn vascular access models [21] and reduces vascular access time during infant resuscitation [22]. We used IO to a greater extent in paediatric than in adult patients. Our results support the recommendation of intraosseous access as the primary choice for vascular access during the resuscitation of children under two years of age. In older children and adults, the IO technique should be reserved as a rescue technique.

The use of IO as a bridging technique, either pre-hospital or in the emergency department, has recently been described [23]. IO can facilitate speedier administration of medication, blood or fluids, thereby increasing patient safety (even after arriving at the hospital) [23,24].

Failed IO access was mainly due to insertion-related problems, with technical problems and extravasation as the most frequent causes. The local fracture experienced using the B.I.G^® ^has also been reported by others [25,26]. Few registered complications in our study may indicate that intraosseous access is a reasonably safe rescue method considering the circumstances in which it is used. However, infections may develop later during treatment, but none were found during follow-up despite non-sterile insertion conditions.

The proximal tibia was the dominant site chosen for intraosseous access, due to the advantage that it does not interfere with ongoing cardiopulmonary resuscitation [1,27].

The most important clinical implications of newer powered devices for IO access relate to critically ill paediatric patients and emergency department resuscitations as a bridging technique when intravenous access cannot readily be achieved. Rates of success on first attempt are important when comparing different techniques. Structured mandatory training in this rescue technique must be emphasised [28].

## Conclusions

Newer intraosseous techniques may enable faster and more reliable vascular access. This can lower the threshold for using intraosseous access techniques on both adult and paediatric patients in critical situations. We believe that all emergency services that handle critically ill or injured patients should be familiar with intraosseous techniques. Further studies are warranted to establish the role of intraosseous access as an emergency rescue technique.

## List of abbreviations

IO: Intraosseous; HEMS: Helicopter Emergency Medical Service; SIDS: Sudden Infant Death Syndrome; IV: Intravenous; EMS: Emergency Medical Service.

## Competing interests

The authors declare that they have no competing interests.

## Authors' contributions

GAS and JKH conceived the study and participated in its design and coordination, and in drafting the manuscript. BEH participated in the design of the study and the statistical analysis, and participated in drafting the manuscript. BHV participated in the design of the study, and the drafting of the manuscript and tables and figures. All authors have read and approved the final manuscript.

## Affiliations

All the authors are employed at the regional university hospital (Haukeland University Hospital), which is part of a national health trust. This study received no external financial support or grants.
